# Quality Assessment of *Gentiana rigescens* from Different Geographical Origins Using FT-IR Spectroscopy Combined with HPLC

**DOI:** 10.3390/molecules22071238

**Published:** 2017-07-24

**Authors:** Zhe Wu, Yanli Zhao, Ji Zhang, Yuanzhong Wang

**Affiliations:** 1 Institute of Medicinal Plants, Yunnan Academy of Agricultural Sciences, Kunming 650200, China; 15887843024@163.com (Z.W.); yanli034120144@126.com (Y.Z.); zjyaas@126.com (J.Z.); 2 Yunnan Technical Center for Quality of Chinese Materia Medica, Kunming 650200, China; 3 College of Traditional Chinese Medicine, Yunnan University of Traditional Chinese Medicine, Kunming 650500, China

**Keywords:** FT-IR spectroscopy, qualitative, partial least squares discriminant analysis, quantitative, support vector machines regression, *Gentiana rigescens*

## Abstract

*Gentiana rigescens* is a precious herbal medicine in China because of its liver-protective and choleretic effects. A method for the qualitative identification and quantitative evaluation of *G. rigescens* from Yunnan Province, China, has been developed employing Fourier transform infrared (FT-IR) spectroscopy and high performance liquid chromatography (HPLC) with the aid of chemometrics such as partial least squares discriminant analysis (PLS-DA) and support vector machines (SVM) regression. Our results indicated that PLS-DA model could efficiently discriminate *G. rigescens* from different geographical origins. It was found that the samples which could not be determined accurately were in the margin or outside of the 95% confidence ellipses. Moreover, the result implied that geographical origins variation of root samples were more obvious than that of stems and leaves. The quantitative analysis was based on gentiopicroside content which was the main active constituent in *G. rigescens.* For the prediction of gentiopicroside, the performances of model based on the parameters selected through grid search algorithm (GS) with seven-fold cross validation were better than those based on genetic algorithm (GA) and particle swarm optimization algorithm (PSO). For the SVM-GS model, the result was satisfactory. FT-IR spectroscopy coupled with PLS-DA and SVM-GS can be an alternative strategy for qualitative identification and quantitative evaluation of *G. rigescens*.

## 1. Introduction

Herbal products, a complementary and alternative therapy, are increasingly gaining popularity in daily life and health care all over the world [1]. In the Western world, herbal medicine is mainly applied in promoting health and treatment of chronic diseases. It also plays a crucial role in multi-component therapeutics [2]. With the increasing usages of herbal medicine, the need for quality control has also increased. Currently, the regulations and pharmacovigilance about herbal medicines are still incomplete and need to be enhanced and improved [1,3]. The issues of quality control such as lack of safety and efficacy in herbal medicine are worthy of attention, because of the lack of reliable, fast and simple technical methods for the quality analysis of herbal medicines [4,5].

*Gentiana rigescens* (family Gentianaceae) is a precious and highly appreciated Chinese herbal medicine, which is widely distributed in the southwest of China, especially in Yunnan Province [6]. As a perennial herb, the root and rhizome are used as the primary medicinal part. This medicine mainly contains iridoids, lignans, triterpenes and others [7]. Among them, gentiopicroside, which belongs to the iridoid class of compounds, is the main active constituents of *G. rigescens* and it is recorded in Chinese Pharmacopoeia (version 2015) as a quality criterion [8]. This compound has long been used in the treatment of hepatic and cholalic diseases, as it has liver-protective and choleretic functions [9].

To our best knowledge, there are many external factors which can influence the quality of herbal medicines, such as geographical origin, harvest time, processing methods, etc. [10,11,12]. According to Yu et al. [13], traditional Chinese medicines and constitutional medicines from China, Japan and Korea differ due to geographical, social environment and other factors. The secondary metabolite composition of herbal medicines varies due to different geographical factors [14,15]. For example, Xia et al. [16] found that phenylalanine, tryptophan, chlorogenic acid syringin and lobetyolin levels in *Codonopsis lanceolata* samples were different depending on the geographical origin and harvesting time. Therefore, with its wide spectrum of therapeutic properties, it is crucial to provide guidance for the quality control of *G. rigescens*.

The conventional analytical methods for qualitative and quantitative analysis usually require operative skills, experience and are labor-intensive in addition to involving organic solvents for sample preparation. In this research, FT-IR spectroscopy, which is fast, clean and cost-effective, was developed to obtain chemical information about *G. rigescens*. It can provide qualitative information about the molecular structure of the components in *G. rigescens* with little or no sample pretreatment [17,18]. In addition, FT-IR spectroscopy, as a powerful analytical technique, has been widely used in the field of qualitative identification and quantitative evaluation in Chinese herbal medicines [19,20]. For these studies, FT-IR spectroscopy combined with multivariate analysis techniques has been applied to identify *G. rigescens* from different geographical origins and determine the iridoids in *G. rigescens*, and the results showed that FT-IR spectroscopy was suitable to provide qualitative and quantitative analyses of *G. rigescens* [21,22]. Similarly, Qi et al. [23], developed a HPLC and FTIR quantitative and qualitative analysis method to distinguish *G. rigescens* samples from different parts and cultivation years.

The objective of this study was to provide an efficient, easy-to-operate and non-hazardous alternative to evaluate the quality variation in different parts of *G. rigescens* from Dali, Lijiang, Diqing and Yuxi in Yunnan Province. Therefore, a method for the qualitative and quantitative analysis of *G. rigescens* has been developed employing FT-IR spectroscopy and chemometrics methods such as partial least squares discriminant analysis (PLS-DA) and support vector machines (SVM) regression. The results of gentiopicroside content determined by high performance liquid chromatography (HPLC) have been used as reference data to build our quantitative analysis model.

## 2. Result and Discussion

### 2.1. HPLC Analysis

All 179 samples were quantified by the HPLC method. Prior to sample determination, the methodology was validated by measuring the stability, repeatability and recovery based on the previous work in our laboratory [24]. The linear relationship of the peak areas and standards of gentiopicroside was y = 7975.52946x + 25.05267, and the correlation coefficient was 0.9999. Therefore, the HPLC method could be considered an accurate and dependable method for measuring gentiopicroside content in *G. rigescens.*

**Figure 1:**
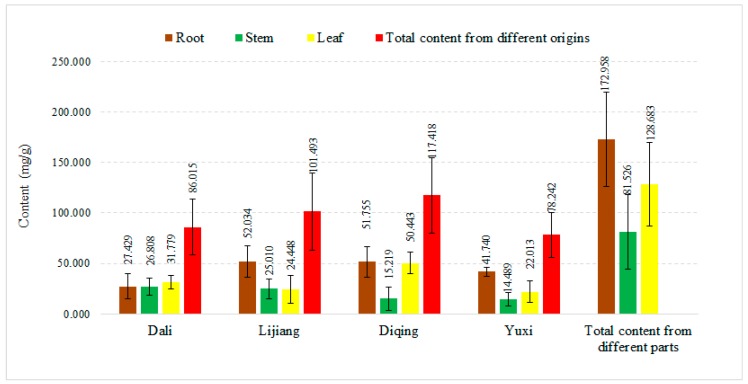
Contents of gentiopicroside in *G. rigescens* (mg/g) with different parts of plants from different geographical origins by HPLC.

Figure 1 shows the average contents of gentiopicroside in different parts of *G. rigescens* from different geographical origins. For the date it can be concluded that samples from Diqing have the greatest gentiopicroside content, followed by those from Lijiang, Dali and Yuxi. Except for the samples from Dali which had the highest gentiopicroside content in leaf, the other three sources showed the highest abundance of gentiopicroside in the roots. It was thus found that not only in the same parts from different geographical origins but also the same part from different geographical origins, the content of gentiopicroside varies greatly. In addition, all samples conformed to the quality standards in the Chinese Pharmacopoeia except the stems from Yuxi (the content of gentiopicroside should be higher than 1.5%).

The above results show that *G. rigescens* samples show a great dependence on geographical origin, which might be influenced by the conditions of these geographical origins. For example, Yuxi is in the central part of Yunnan Province which is mainly a subtropical area, while the others are in the northwest of Yunnan Province, which belongs to the temperate climate zone area [25]. This indicates that the quality of the herb showed geographical and habitat dependences to some extent. Similar results have been reported for the quality of *Paris* from different geographic origins [26].

### 2.2. FT-IR Spectral Features

**Figure 2:**
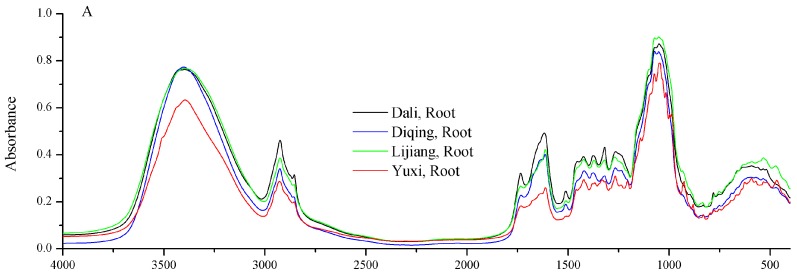

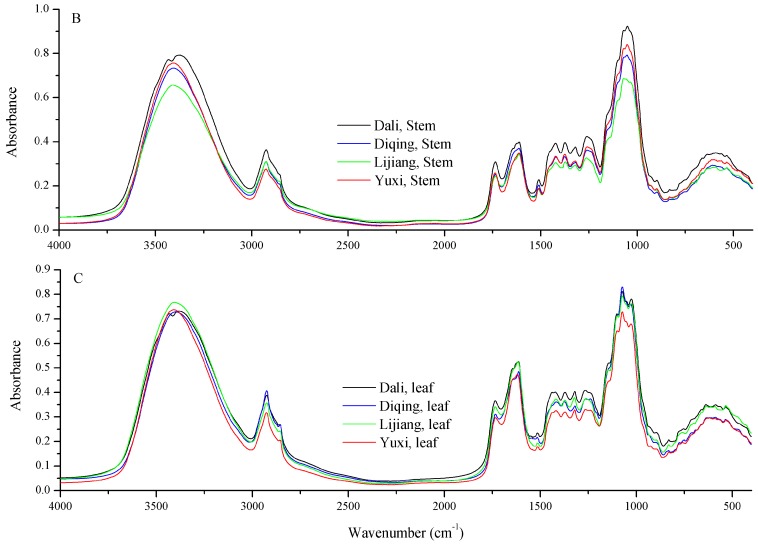
The average FT-IR spectra of root (**A**), stem (**B**) and leaf (**C**) in *G. rigescens* from different geographical origins (Dali, Lijiang, Diqing and Yuxi) in the 4000–400 cm^−1^ range.

The average 4000–400 cm^−1^ FT-IR spectra of different parts of *G. rigescens* from different geographical origins are shown in Figure 2. On the whole, there is no distinct difference among the average FT-IR spectra, which overlap. However, the absorption intensities of the average FT-IR spectra vary a lot. Compared to other geographical origins, the absorption intensity is obviously lower in the root sample of Yuxi (Figure 2A). A broad absorption band is found at around 3399 cm^−1^, which is due to the O–H stretch. The bands at 2933 and 2856 cm^−1^ are CH_3_ asymmetric stretching and stretching vibration of esters, respectively. The peak around 1937 cm^−1^ is assigned to the C=O stretching vibration of acid amides [27]. In addition, the intense absorption peaks in 1070 and 1619 cm^−1^ are the main absorption bands of iridoids or glycosides, which correspond to C–O or C–O–C stretching and C–C asymmetric stretching vibrations [28,29]. According to studies of *G. rigescens* by Mi et al. [29] and Yang et al. [30], the active compounds gentiopicroside, swertiamarin, and chiratin and other iridoids in *G. rigescens* all contain C–O or C–O–C and C–C bonds.

### 2.3. Multivariate Analysis

In the PLS-DA and SVM regression models, the samples were divided into two categories: training set and validation set. Two-thirds of the samples were classified as training set and the others were assigned to the validation set by the Kennard-Stone algorithm [31].

#### 2.3.1. PLS-DA Models

**Figure 3:**
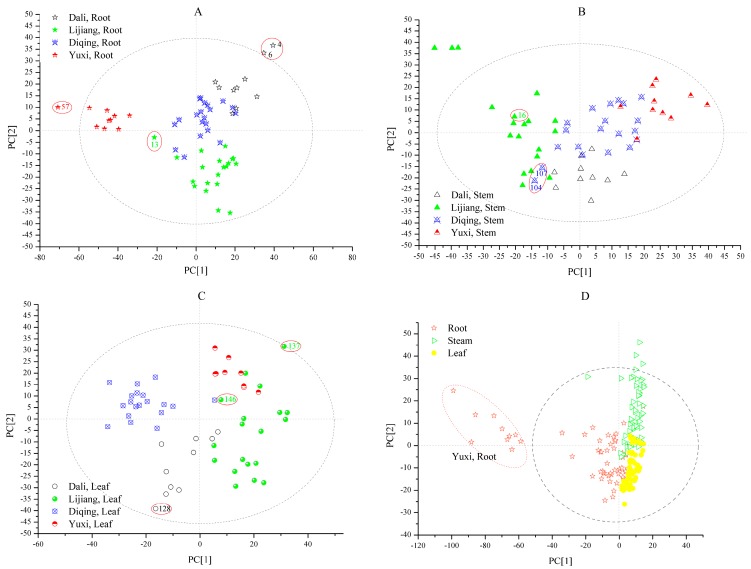
The scatter plot of PLS-DA FT-IR spectra display the information of samples of root (**A**), stem (**B**), leaf (**C**) and three parts (root, stem and leaf) (**D**) in *G. rigescens* from different geographical origins (Dali, Lijiang, Diqing and Yuxi). The abscissa represents the variation of the first component and the ordinate represents the variation of the second component.

Four PLS-DA models were built: roots from different geographical origins (model 1), stems from different geographical origins (model 2), leafs from different geographical origins (model 3) and three different parts (root, stem and leaf) (model 4). In order to solve the problem of band overlap, baseline drift and noise, spectral preprocessing was applied [32,33,34]. For the PLS-DA models 1, 2, 3 and 4, the optimized spectral preprocessing were second derivation (2Der), multiplicative scatter correction (MSC) + 2Der, standard normal variate (SNV) + 2Der and MSC + 2Der, respectively. After optimized spectral preprocessing of the FT-IR spectra, the PLS-DA models were established by the first two principal components (PC1 and PC2) for qualitative analysis of all *G. rigescens* samples (Figure 3)*.*

Figure 3A displays the score plot of *G. rigescens* roots from Dali, Lijiang, Diqing and Yuxi. In the score plot, samples from different geographical origins can be clustered in a range and distinguished from others. Samples from Yuxi are far away from the other three geographical origins, while the distance of the other three geographical origins are closer. It can be seen that PC1 separates the samples of Yuxi from others, the former is in the central of Yunnan Province and the latter is in the northwest of Yunnan Province, which matches the regularities of gentiopicroside content distribution analyzed by HPLC. PC2 separates the samples of Diqing and Dali from samples of Lijiang.

The prediction results of the model parameters determination coefficient (R^2^), root-mean-square error of estimation (RMSEE) and root-mean-square error of cross validation (RMSECV) are listed in Table 1. The first six principal components (96.0%) are employed for model 1. The R^2^ is greater than 0.94 and the RMSEE and RMSECV are low, which are less than 0.25. Thereinto, model 1 of samples from Yuxi have the best performance with the highest R^2^ and lowest RMSEE and RMSECV. As seen in Table 2, according to the Galtier criterion, all the samples are identified correctly except the four samples numbered 4, 6, 13 and 57. Sample 13 from Lijiang was misidentified as coming from Diqing, and the three other samples (4, 6 and 57) can’t be judged accurately. More interestingly, the uncertain samples are all outside of the 95% confidence ellipses in the scatter plot (Figure 3A). The prediction accuracy of model 1 is 80%.

The score plot of *G. rigescens* stems from Dali, Lijiang, Diqing and Yuxi is described in Figure 3B. The *G. rigescens* stems from different geographical origins can be separated except for a few that were mixed. The stems from Lijiang are distributed widely, while the samples from the other three origins are centrally distributed. It is shown that PC1 separates the samples of Lijiang and Yuxi from those of Dali and Diqing while PC2 separates the samples of Dali from those from Diqing.

Table 1 shows the parameters of R^2^, RMSEE and RMSECV in model 2. The first four principal components are employed for model 2, and the cumulative contribution reached 88.5%. The model of samples from Yuxi have the best precision, with high R^2^ (0.9472) and low RMSEE (0.0877) and RMCECV (0.1689).

According to the Galtier criterion, two samples from Diqing (104 and 107) are identified as Lijiang samples erroneously and sample 76 can’t be judged accurately (Table 3). As seen in Figure 3B, samples 104 and 107 are close to the samples from Lijiang which is the same as the results from Table 3. The prediction accuracy of model 2 is 85%.

Figure 3C displays the score plots of *G. rigescens* leaves from Dali, Lijiang, Diqing and Yuxi. The cumulative contribution reached 90.2%, when the first four principal components are employed. In Figure 3C, the samples from Dali, Diqing and Yuxi can be clustered into three groups, while Lijiang samples are distributed dispersedly. The samples from Diqing and Dali can be separated from the Yuxi and Lijiang ones by PC1. In addition, the samples from Yuxi and Lijiang can be distinguished by PC2.

The R^2^, RMSEE and RMSECV of model 3 are shown in Table 1. The performances of the different geographical origin discrimination are good, with high R^2^ (>0.88) and low RMSEE (<0.17) and RMSECV (<0.20). Thereinto, the best performance is for the samples from Yuxi. In Table 4, the samples 128 and 137 can’t be confirmed. Moreover, a sample from Lijiang (146) is judged as a Dali sample by mistake. More interestingly, the uncertain samples are in the margin of the 95% confidence ellipses (Figure 3C) like the result of model 1. The prediction accuracy of model 3 is 85%.

The score plot (Figure 3D) shows that all *G. rigescens* samples can be separated based on three parts (root, stem and leaf). The first four principal components which represent 87.0% of the explained variance are applied to model 4. It is clear that the roots, stems and leaves can be separated completely. Thia indicates that the metabolic profiles of different parts in *G. rigescens* are unlike. The stems and leaves samples cluster in two concentrated regions and the distance between them is close, however, the root samples distribute dispersedly whereby the root samples from Yuxi cluster outside the 95% confidence ellipses and the distance between the roots samples from Yuxi and the other root samples are large (Figure 3D). This indicates that the geographical origins variation of root samples are more obvious than those of stems and leaves. Moreover, it is shown that PC1 separates the root samples from stem and leaf samples and PC2 separates the stem samples from leaf samples.

As shown in Table 1, the best performance of model 4 is leaf samples, followed by stem and root. The prediction accuracy of model 3 is 86.7%, which six samples (29, 51, 61, 62, 106 and 111) are uncertain whether class belongs to, and three stem samples (63, 78 and 83) are regarded as root samples (Table 5). The result shows that the metabolic profiles of leaf samples may be similar to root samples.

#### 2.3.2. SVM Regression Model

After optimized spectral preprocessing by orthogonal signal correction (OSC) and 2Der, all data was normalized in the region between 1 and 2. Then, the parameters c and g in the SVM regression model were selected by a grid search algorithm (GS) with seven-fold cross validation, genetic algorithm (GA) and particle swarm optimization algorithm (PSO). The GS with cross-validation can prevent the problem of overfitting and can be easily parallelized on account of parameters (c and g) [35]. The algorithm of GA is based on the principle of survival of the fittest. In GA algorithm, the most obvious superiority is that it can find the optimal or near the optimal solutions in the relatively low computation [36]. The basic concept of PSO algorithm is derived from the study of bird predation behavior. It is a new stochastic optimization algorithm based on the intelligent [37]. Finally, the best parameters were used to train the training set.

**Figure 4:**
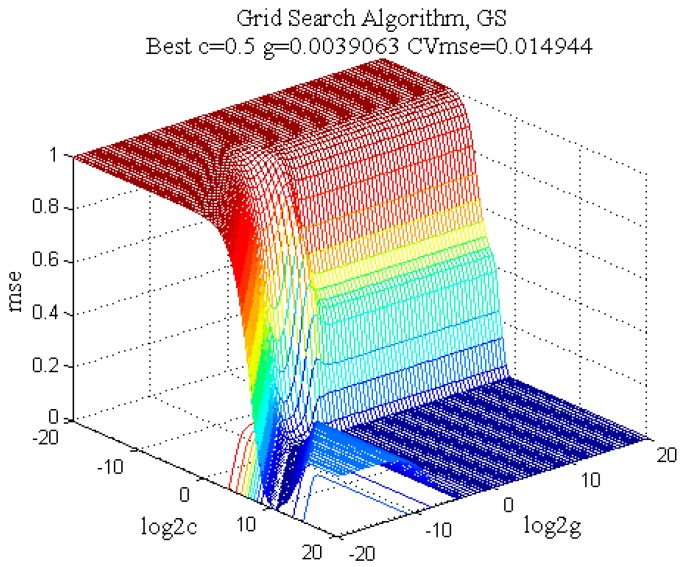
The 3D view of the optimization results for parameters c and g by grid search method with seven-fold cross validation.

**Figure 5:**
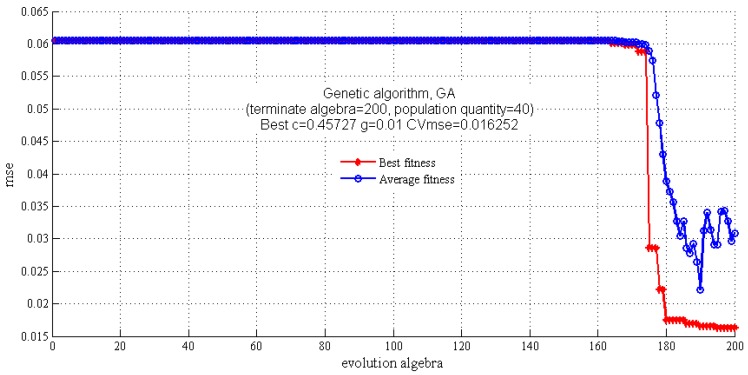
The optimization results for parameters c and g by genetic algorithm.

**Figure 6:**
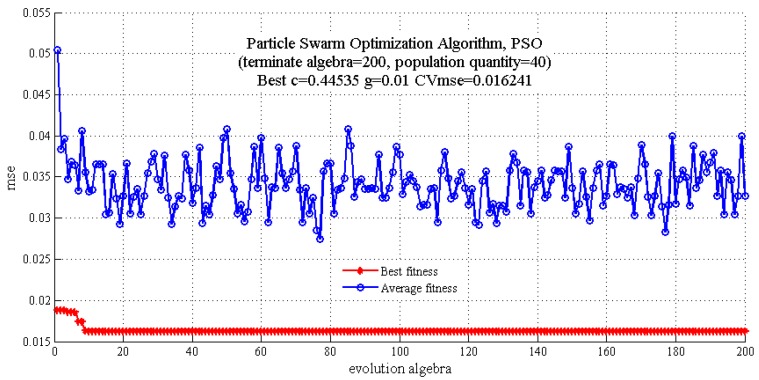
The optimization results for parameters c and g by particle swarm optimization algorithm.

In this study, the GS algorithm was applied to screen the parameters c and g in the region of 1 to 2^20^ and 2^−20^ to 1, respectively. As can be seen in Figure 4, the results of c, g and cross-validation mean square error (CVmse) which are calculated by the GS algorithm are 0.5, 0.0039 and 0.0149, respectively. In addition, the terminate algebra was set as 200 and population quantity was set as 40 in the GA algorithm. It is shown that the optimum parameters c, g and CVmse are 0.4572, 0.01 and 0.0163, respectively (Figure 5). Finally, the PSO algorithm was also applied to select the parameters and the detail parameter (terminate algebra and population quantity) of PSO was the same as the GA algorithm (Figure 6). The results of the PSO algorithm are as follows: c = 0.4453, g = 0.01 and CVmse = 0.01624. The aforementioned algorithms were all applied for building the SVM regression models.

Table 6 shows the performances achieved by the GS, GA and PSO SVM regression models for predicting the content of gentiopicroside. From Table 6, it is observed that the highest R_t_^2^ (96.39%) and RMSEE (3.1056) for training set and the highest R_v_^2^ (83.57%) and the lowest RMSEP (11.1421) for validation set is obtained by the GS algorithm. Therefore, the GS method gives the best performance for the prediction of gentiopicroside content in *G. rigescens.*

**Figure 7:**
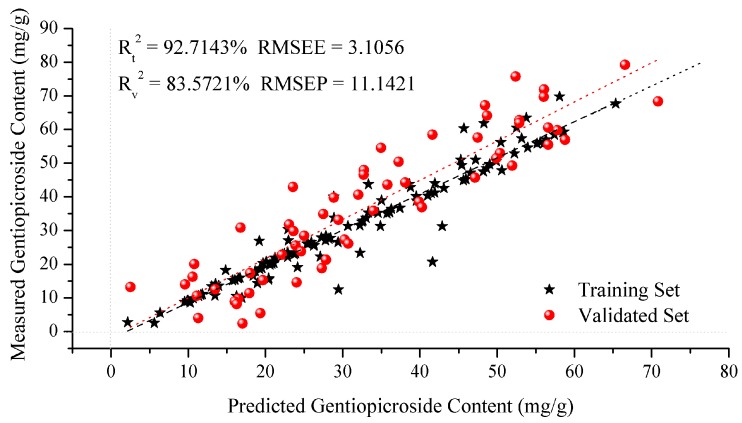
Correlation diagram between FT-IR predicted values and the reference values in the training and validation sets for gentiopicroside.

Figure 7 shows the result of validation set for prediction vs. measured gentiopicroside content which was achieved by the GS algorithm in the SVM regression model. The samples from the training and validation sets are all symmetrically distributed on the both sides of the regression line. Satisfactory predictions with R_t_^2^, R_t_^2^, RMSEE and RMSEP are achieved and good agreement with the SVM regression model built for predicting gentiopicroside content in *G. rigescens* is observed.

## 3. Materials and Methods

### 3.1. Plant Materials and Reagents

Wild fresh *G. rigescens* samples were collected from Dali, Lijiang, Diqing and Yuxi in Yunnan Province, China (Table 7). Specimens were identified by Prof. Jinyu Zhang (Institute of Medicinal Plants, Yunnan Academy of Agricultural Sciences).

Potassium bromide (KBr) was purchased from Tianjin Fengchuan Fine Chemical Research Institute (Tianjin, China). A gentiopicroside standard was supplied by the National Institute for the Control of Food and Drug control (Beijing, China). Analytical grade methanol (80%, *v*/*v*) used as extraction solvent was obtained from Xilong Chemical Company (Guangdong, China). Chromatography grade acetonitrile and formic acid were purchased from Sigma-Aldrich (Flanders, NJ, USA).

### 3.2. Sample Preparation

The *G. rigescens* samples were divided into three parts (root, stem and leaf) after cleaning and dried for 24 h at 60 °C. Then, all samples were powdered in a high-speed blender and passed through an 80 mesh stainless steel sieve, separately. Then, the sieved powders were kept in Ziploc bags and stored at room temperature prior to analysis.

A sample (25 mg) of each powder was weighed accurately using an electronic balance (XS125A, Precisa, Basel, Switzerland) and soaked with 1.5 mL of 80% methanol for 30 min under ultrasonication at room temperature. Before analysis by HPLC, the extracts were filtered through a 0.22 μm membrane filter (Millipore, Bedford, MA, USA). All the extracts of *G. rigescens* samples were subjected to analysis.

### 3.3. HPLC Conditions

Gentiopicroside was determined using an Agilent 1260 Infinity system (Agilent Technologies, Palo Alto, CA, USA) equipped with a G1311 diode array detector, a quaternary pump and an on-line degasser. An Agilent Zorbax AB-C18 column (5 µm, 4.6 × 250 mm) was utilized for the chromatographic separations. The mobile phase consisted of 0.1% aqueous formic acid in water (A) and acetonitrile (B). The gradient elution procedure was as follows: the initial mobile phase composition was set to 5% B for 5 min, then increased stepwise linearly first to 10% B from 5 to 10 min, then to 26% B from 10 to 26 min, and finally decreased to 30% for 30 min. The column temperature was maintained at 30 °C. The flow rate was set at 0.3 mL/min and the injection volume was 10 µL. During the experiment, the detective wavelength was set at 241 nm. Every samples were detected three times, and the averaged spectra were employed for further analysis.

### 3.4. FT-IR Spectra Acquisition

FT-IR spectra acquisition was performed by using a Fourier transform infrared spectrometer (Perkin Elmer, Norwalk, CT, USA) equipped with a deuterated triglycine sulfate detector. Powdered samples (1.2 mg) and 100 mg of KBr were precisely weighed and mixed evenly. Then, the mixed powder was pressed into a tablet by using a table press (YP-2, Shanghai Shanyue Instrument Inc., Shanghai, China) for detection. Each FT-IR spectrum was collected in the region of 4000–400 cm^−1^ with a resolution of 4 cm^−1^ and a total of 16 co-added scans. Pure KBr spectra were recorded as background spectra for deducting the CO_2_ and H_2_O peaks in real-time. Each spectrum was scanned in triplicate under constant temperature (25 °C) and humidity conditions, and the averaged spectra were employed for further analysis.

### 3.5. Multivariate Data Analysis

Before analysis, two-thirds of the samples were classified as the training set and the others were assigned to the validation set using the Kennard-Stone algorithm. Qualitative and quantitative models were developing with PLS-DA and SVM regression, respectively. PLS-DA, a supervised analysis method, was successfully applied to the classification of the FT-IR spectra [38]. The basic principle of PLS-DA was to reduce the independent variables X for obtaining a maximum covariance between X and Y variables [39,40]. SVM, a state-of-the-art method of classification and regression technique, was proposed on the basis of statistical learning theory by Vapnik [41]. The fundamental objective of SVM was to construct a separating plane that all the data points have the shortest distance to [42]. SVM is famous for its advantages which avoid over-fitting problems and improve the generalization and accurate prediction ability by introducing a structure risk function. Rather than empirical risk that minimizes the misclassification errors in the training set, structural risk minimizes the misclassification error on a settled but unware probability distribution in which previously invisible data points are drawn at random [43,44]. Moreover, SVM can effectively overcome the “curse of dimensionality” by introducing a kernel function. SVM thus successfully solves non-linear prediction problems [45].

In this paper, a library for the SVM toolkit LIBSVM 3.21 was applied in data processing which was developed by Chang and optimized by Lin [46]. The performance of SVM depends mainly on the type of kernel function and its parameters [47]. There are four kinds of kernel function types in this toolkit, including: linear, radial basis function (RBF), polynomial and sigmoid. Usually, RBF is selected to build the regression models for prediction [42,48,49]. The error penalty parameter c and RBF kernel parameter g are the major parameters in the SVM model with RBF [50]. The RBF kernel parameter g, as kernel width, had an impact on the prediction of the SVM model, while c controls the complexity of the SVM model.

### 3.6. Evaluation of Model Performance

The determination coefficient (R^2^), root-mean-square error of estimation (RMSEE), root-mean-square error of cross validation (RMSECV) and root-mean-square error of prediction (RMSEP) were considered to evaluate the performance of qualitative and quantitative model.

R^2^ (Equation (1)) is the correlation between the measured values and predicted values. Generally, a higher R^2^ (<1) value means a better performance of both kinds of models [51]:

(1) $$R^{2}=\sum_{i=1}^{N}{\left(y_{i}-{\hat{y}}_{i}\right)}^{2}/\sum_{i=1}^{N}{\left({\bar{y}}_{i}-\bar{y}\right)}^{2}$$

where, y_i_ is the measured value while ŷ_i_ is the predicted value. $\bar{y}$ is the mean value, and N is the number of samples.

RMSEE, RMSECV and RMSEP were applied to evaluate the precision of model performance (Equations (2)–(4)). The lower RMSEE, RMSECV and RMSEP are, the fitter the models obtained [52,53,54]. Moreover, the robustness of models depends on the difference between the determination coefficient for the training set ($R_{v}^{2}$) and the determination coefficient for the validation set ($R_{v}^{2}$). The smaller the difference between them, the more satisfactory the model [55]:

(2) $$\mathrm{RMSEE}=\sqrt{\frac{\sum_{i=1}^{N_{t}}{\left({\hat{y}}_{i}-y_{i}\right)}^{2}}{N_{t}-1}}$$

(3) $$\mathrm{RMSECV}=\sqrt{\frac{\sum_{i=1}^{N_{t}}{\left({\hat{y}}_{i}-y_{i}\right)}^{2}}{N_{t}}}$$

(4) $$\mathrm{RMSEP}=\sqrt{\frac{\sum_{i=1}^{N_{v}}{\left({\hat{y}}_{i}-y_{i}\right)}^{2}}{N_{v}}}$$

where, N_t_ is the number of the training set and N_v_ is the number of validation set. In addition, in the qualitative model, the classification accuracy of the validation set depends on the predicted value (Y_pre_), and deviation values (Y_dev_) which are based on the following standards: (1) when Y_pre_ > 0.5 and Y_dev_ < 0.5, the sample of validation set belongs to the class; (2) when Y_pre_ < 0.5 and Y_dev_ < 0.5, the sample of validation set does not belong to the class; (3) when Y_dev_ > 0.5, it means that the sample can’t judge accurately whether it belongs to the class or not [56,57].

### 3.7. Software

The FT-IR spectra were processed using Omnic (Version 8.2, Thermo Fisher Scientific, Madison, WI, USA). The chromatographic fingerprints were conducted using the Similarity Evaluation System for Chromatographic Fingerprints of traditional Chinese Medicines (Version 2004a, Chinese Pharmacopoeia Commission, Beijing, China). The PLS-DA models were created by Simca (Version 13.0, Umetrics, Umea, Sweden), while the MSV regression model was created by MATLAB (Version R2014a, MathWorks, Natick, MA, USA) with the LIBSVM-Faruto toolkit (Version Ultimate 3.1M) [58]. All the figures were drawn by Origin (Version 8.0, Originlab, North Hampton, MA, USA) and MATLAB.

## 4. Conclusions

In this study, a rapid FT-IR spectroscopic method combined with a chemometrics procedure was developed for the qualitative and quantitative analysis of *G. rigescens*. The discrimination of different parts of *G. rigescens* plants from different geographical origins by using FT-IR spectroscopy in combination with PLS-DA was presented. The feasibility of rapid quantitative analysis of gentiopicroside content in *G. rigescens* by application of FT-IR spectroscopy in combination with SVM regression was investigated. The results showed that for gentiopicroside determination, the parameter selection method of GS of a SVM regression model provided a good prediction. Overall, FT-IR spectroscopy combined with chemometrics could be a promising method for quality assessment of *G. rigescens*.

## Figures and Tables

**Table 1:** Statistics of the optimal calibration models.

Types Parameter	Model 1				Model 2				Model 3				Model 4		
	Dali, Root	Diqing, Root	Lijiang, Root	Yuxi, Root	Dali, Stem	Diqing, Stem	Lijiang, Stem	Yuxi, Stem	Dali, Leaf	Diqing, Leaf	Lijiang, Leaf	Yuxi, Leaf	Root	Stem	Leaf
R^2^	0.9543	0.9654	0.9426	0.9762	0.8346	0.8464	0.9098	0.9472	0.9031	0.8964	0.8854	0.9231	0.8334	0.8425	0.9331
RMSEE	0.0852	0.0985	0.1247	0.0615	0.1552	0.1999	0.1531	0.0877	0.1364	0.1695	0.1464	0.1140	0.1835	0.1927	0.1295
RMSECV	0.1785	0.2313	0.1992	0.0709	0.2740	0.2688	0.1807	0.1689	0.1614	0.1924	0.1680	0.1713	0.2272	0.2304	0.1445

Model evaluation statistics for partial least squares discriminant analysis (PLS-DA) models. R^2^: determination coefficient; RMSEE: root-mean-square error of estimation; RMSECV: root-mean-square error of cross-validation.

**Table 2:** Results of the PLS-DA model 1 validation set samples.

Samples	Actual Class	Calculated Class	Y_Pre_	Y_dev_
2	Dali, Root	Dali, Root	1.1112	0.4306
4	Dali, Root	Uncertain	1.5243	0.6372
6	Dali, Root	Uncertain	1.5774	0.6637
7	Dali, Root	Dali, Root	1.2030	0.4765
13	Lijiang, Root	Diqing, Root	0.4190	0.1300
14	Lijiang, Root	Lijiang, Root	0.6108	0.2930
18	Lijiang, Root	Lijiang, Root	0.8904	0.3202
20	Lijiang, Root	Lijiang, Root	0.8029	0.3449
21	Lijiang, Root	Lijiang, Root	1.1986	0.4743
24	Lijiang, Root	Lijiang, Root	1.0592	0.4046
25	Lijiang, Root	Lijiang, Root	0.8340	0.2920
31	Diqing, Root	Diqing, Root	0.7014	0.2522
40	Diqing, Root	Diqing, Root	0.8934	0.3217
42	Diqing, Root	Diqing, Root	0.7153	0.2364
44	Diqing, Root	Diqing, Root	0.7702	0.2601
45	Diqing, Root	Diqing, Root	1.0444	0.3972
47	Diqing, Root	Diqing, Root	0.8626	0.3079
54	Yuxi, Root	Yuxi-Root	0.9195	0.3351
57	Yuxi, Root	Uncertain	1.3332	0.5416
59	Yuxi, Root	Yuxi-Root	1.1181	0.4341

Y_pre_: predicted value; Y_dev_ and deviation values.

**Table 3:** Results of the PLS-DA model 2 validation set samples.

Samples	Actual Class	Calculated Class	Y_Pre_	Y_dev_
61	Dali, Stem	Dali, Stem	0.9741	0.4110
62	Dali, Stem	Dali, Stem	1.1943	0.4722
66	Dali, Stem	Dali, Stem	0.8053	0.2776
70	Dali, Stem	Dali, Stem	0.5274	0.1387
72	Lijiang, Stem	Lijiang, Stem	1.0518	0.4318
74	Lijiang, Stem	Lijiang, Stem	0.9547	0.3524
76	Lijiang, Stem	Uncertain	1.3367	0.6355
81	Lijiang, Stem	Lijiang, Stem	0.7931	0.2796
87	Lijiang, Stem	Lijiang, Stem	1.0656	0.4078
88	Lijiang, Stem	Lijiang, Stem	1.0032	0.3766
91	Diqing, Stem	Diqing, Stem	1.2258	0.4879
94	Diqing, Stem	Diqing, Stem	1.1724	0.4612
95	Diqing, Stem	Diqing, Stem	1.0654	0.4077
104	Diqing, Stem	Lijiang, Stem	0.4763	0.2348
105	Diqing, Stem	Diqing, Stem	0.6590	0.2045
107	Diqing, Stem	Lijiang, Stem	0.3148	0.2175
112	Yuxi, Stem	Yuxi, Stem	0.9311	0.3704
117	Yuxi, Stem	Yuxi, Stem	0.9702	0.3601
120	Yuxi, Stem	Yuxi, Stem	0.9334	0.3417

Y_pre_: predicted value; Y_dev_ and deviation values.

**Table 4:** Results of PLS-DA model 3 validate set samples.

Samples	Actual Class	Calculated Class	Y_Pre_	Y_dev_
123	Dali, Leaf	Dali, Leaf	0.7455	0.2478
128	Dali, Leaf	Uncertain	1.4334	0.5917
133	Lijiang, Leaf	Lijiang, Leaf	0.8984	0.3242
134	Lijiang, Leaf	Lijiang, Leaf	0.6928	0.2214
135	Lijiang, Leaf	Lijiang, Leaf	0.5073	0.1949
136	Lijiang, Leaf	Lijiang, Leaf	0.8768	0.3134
137	Lijiang, Leaf	Uncertain	1.1790	0.6497
139	Lijiang, Leaf	Lijiang, Leaf	0.9106	0.3494
140	Lijiang, Leaf	Lijiang, Leaf	0.5083	0.1241
141	Lijiang, Leaf	Lijiang, Leaf	0.9160	0.3330
144	Lijiang, Leaf	Lijiang, Leaf	1.0707	0.4103
145	Lijiang, Leaf	Lijiang, Leaf	1.3312	0.5406
146	Lijiang, Leaf	Dali, Leaf	0.3026	0.1056
154	Diqing, Leaf	Diqing, Leaf	1.1157	0.4329
160	Diqing, Leaf	Diqing, Leaf	0.8556	0.3242
165	Diqing, Leaf	Diqing, Leaf	0.5760	0.1630
166	Diqing, Leaf	Diqing, Leaf	1.1611	0.4555
170	Yuxi, Leaf	Yuxi, Leaf	0.9046	0.3273
176	Yuxi, Leaf	Yuxi, Leaf	0.8712	0.3127
178	Yuxi, Leaf	Yuxi, Leaf	0.9259	0.3379

Y_pre_: predicted value; Y_dev_ and deviation values.

**Table 5:** Results of PLS-DA model 4 validation set samples.

Samples	Actual Class	Calculated Class	YPre	Ydev
5	Root	Root	0.7803	0.3378
6	Root	Root	0.9955	0.4414
7	Root	Root	0.7524	0.2794
12	Root	Root	0.9642	0.4205
13	Root	Root	0.9584	0.4167
14	Root	Root	0.8682	0.3566
15	Root	Root	0.7034	0.2467
18	Root	Root	0.8453	0.3500
19	Root	Root	0.8823	0.3660
20	Root	Root	0.8876	0.3695
21	Root	Root	0.6858	0.2350
22	Root	Root	0.8692	0.3573
24	Root	Root	0.9851	0.4345
29	Root	Uncertain	1.1444	0.5407
30	Root	Root	0.9511	0.4118
31	Root	Root	0.6869	0.2357
32	Root	Root	0.9623	0.4193
34	Root	Root	0.8161	0.3218
38	Root	Root	0.9635	0.4201
40	Root	Root	1.0701	0.4912
41	Root	Root	0.6536	0.2135
43	Root	Root	0.7605	0.2848
49	Root	Root	0.9819	0.4324
50	Root	Root	0.7037	0.3252
51	Root	Uncertain	1.2548	0.6143
52	Root	Root	0.9847	0.4343
53	Root	Root	0.8527	0.3462
57	Root	Root	0.8420	0.3391
58	Stem	Stem	0.5076	0.1588
61	Stem	Uncertain	1.1209	0.5250
62	Stem	Uncertain	1.2384	0.6033
63	Stem	Root	0.2768	0.2470
70	Stem	Stem	0.6177	0.1896
71	Stem	Stem	0.7688	0.2903
73	Stem	Stem	0.6066	0.1822
78	Stem	Root	0.1209	0.2604
82	Stem	Stem	0.7153	0.2596
83	Stem	Root	0.4597	0.0842
84	Stem	Stem	0.6782	0.2299
86	Stem	Stem	1.0495	0.4932
89	Stem	Stem	0.7826	0.2995
104	Stem	Stem	0.7306	0.2648
106	Stem	Uncertain	1.2252	0.5946
108	Stem	Stem	0.8728	0.3596
110	Stem	Stem	0.7044	0.2639
111	Stem	Uncertain	1.5978	0.8430
112	Stem	Stem	1.0158	0.4550
122	Leaf	Leaf	1.0092	0.4505
123	Leaf	Leaf	0.9702	0.4246
129	Leaf	Leaf	0.6900	0.2377
130	Leaf	Leaf	0.9760	0.4285
132	Leaf	Leaf	0.7933	0.3066
133	Leaf	Leaf	0.8667	0.3556
141	Leaf	Leaf	0.8623	0.3526
149	Leaf	Leaf	0.9041	0.3805
153	Leaf	Leaf	0.9990	0.4438
159	Leaf	Leaf	0.8485	0.3434
163	Leaf	Leaf	0.9792	0.4325
165	Leaf	Leaf	1.0081	0.4499
170	Leaf	Leaf	0.9311	0.3985

Y_pre_: predicted value; Y_dev_ and deviation values.

**Table 6:** Statistics of the SVM models.

Model	c	g	CVmse	R_t_^2^ (%)	RMSEE	R_v_^2^ (%)	RMSEP
GS-SVM	0.5000	0.0040	0.0149	92.7143	3.1056	83.5721	11.1421
GA-SVM	0.4573	0.0100	0.0163	96.3977	3.1760	82.3279	11.1504
PSO-SVM	0.4454	0.0100	0.0162	96.3120	3.2131	82.3529	11.1506

R_t_^2^: determination coefficient for training set; R_v_^2^: determination coefficient for validated set; RMSEE: root-mean-square error of estimation; RMSEP: root-mean-square error of prediction.

**Table 7:** Information of *G. rigescens.*

No.	Site	Description	No.	Site	Description	No.	Site	Description
1–10	Dali, Yunnan	Root	61–70	Dali, Yunnan	Stem	121–130	Dali, Yunnan	Leaf
11–30	Lijiang, Yunnan	Root	71–90	Lijiang, Yunnan	Stem	131–149	Lijiang, Yunnan	Leaf
31–50	Diqing, Yunnan	Root	91–110	Diqing, Yunnan	Stem	150–169	Diqing, Yunnan	Leaf
51–60	Yuxi, Yunnan	Root	111–120	Yuxi, Yunnan	Stem	170–179	Yuxi, Yunnan	Leaf
